# Points of Interest (POI): a commentary on the state of the art, challenges, and prospects for the future

**DOI:** 10.1007/s43762-022-00047-w

**Published:** 2022-06-28

**Authors:** Achilleas Psyllidis, Song Gao, Yingjie Hu, Eun-Kyeong Kim, Grant McKenzie, Ross Purves, May Yuan, Clio Andris

**Affiliations:** 1 Faculty of Industrial Design Engineering, Delft University of Technology, Landbergstraat 15, Delft, 2628CE the Netherlands; 2 Department of Geography, University of Wisconsin, Madison, USA; 3 Department of Geography, University at Buffalo, Buffalo, USA; 4 Department of Geography, University of Zurich, Zurich, Switzerland; 5 Department of Geography, McGill University, Montreal, Canada; 6 School of Economic, Political and Policy Sciences, University of Texas at Dallas, Richardson, USA; 7 School of City & Regional Planning and School of Interactive Computing, Georgia Institute of Technology, Atlanta, USA

**Keywords:** Points of Interest, Spatial Data, Geographic Representation, Place

## Abstract

In this commentary, we describe the current state of the art of points of interest (POIs) as digital, spatial datasets, both in terms of their quality and affordings, and how they are used across research domains. We argue that good spatial coverage and high-quality POI features — especially POI category and temporality information — are key for creating reliable data. We list challenges in POI geolocation and spatial representation, data fidelity, and POI attributes, and address how these challenges may affect the results of geospatial analyses of the built environment for applications in public health, urban planning, sustainable development, mobility, community studies, and sociology. This commentary is intended to shed more light on the importance of POIs both as standalone spatial datasets and as input to geospatial analyses.

## Introduction

Points of interest (POIs) are locations that, historically, cartographers have added to maps to communicate an interesting or relevant named place, using cartographic symbols and labels. POIs typically include visually and culturally important features, perhaps also with implications for navigation such as mountain summits, churches, and locations of historical battlefields. Today, we use digital POI datasets to show the locations of these features. Broadly, POIs serve as third places; that is, destinations besides home (the first place) and work (the second place) (Oldenburg, R. (Ed.), 2001). These include parks, pubs, cafés, community centers, and bookstores, among others. Contemporary digital POIs are proxies for real-world locations (e.g., restaurants, theaters, schools), usually represented as geometric point entities. They often denote destinations that support activities like jobs, tourism, recreation, and wayfinding. POIs are an important part of daily life and sustain society. Since POIs are locations with meaning (Tuan, 1977), they can also be referred to and considered to be places.

With increasing digitization, the number of POIs that can be represented in an area is no longer restricted by the physical space available on a map, yielding a larger number of recorded POIs available for mapping and spatial analysis. Sources of POI data have grown in number and diversity over the years, and the spatial information extracted from them has grown in complexity. Social media and digital mapping sources such as Yelp, Foursquare, Google Places, OpenStreetMap, among others, offer new crowdsourced and private sector generated POI datasets. Each source contains longitude-latitude points annotated with various attributes (such as hours of operation or age of building) and thus contributes to a different interpretation of the locations they represent.

Accordingly, this POI data ecosystem encourages us to reflect on how POI data is generated and conflated, who creates these datasets, how POIs are classified and described, what types of events occur at POIs, and how to express the value of POIs. The levels of completeness, accuracy, and consistency regarding (mapped and unmapped) POIs and their accompanying attributes vary across data sources and geographies. Such variation is likely stronger in areas of more deprivation and the global South, where the commercial impetus to add such information is more limited.

We lack a general commentary that encapsulates and reflects on the broad set of human experiences and challenges that derive from the modern POI data ecosystem and its platial implications. Without reflecting critically on who is creating POI data and for what purpose, together with the broader philosophical question of what it means to emphasize certain destination-type features in geographic space, we risk developing knowledge systems that privilege certain perspectives over others.

In this commentary, we aim to describe the state-of-the-art research on POIs in geographic information science (GIScience) and computational urban science, and discuss challenges and considerations when creating and using these datasets. With both data generation and data use in mind, we then outline a research agenda for scientific inquiries, applications, and practices. This commentary is the result of a group discussion at the *Points of Interest (POInt) Research Symposium*, a virtual event sponsored by the Georgia Institute of Technology on April 30th, 2021.^1^ The intended audience of this commentary is those working to create and/or use POI datasets, and, more broadly, those who are interested in (reflecting on) the process of spatial data formation.

The remainder of this commentary is structured as follows: We first list scientific questions related to POIs and then describe data sources and attributes of POIs. Next, we outline indicative research and real-world applications that use POI datasets followed by some challenges related to POIs in terms of data quality and research. We conclude by describing prospects for the future of POI research, data generation, and use.

## Scientific questions related to POIs

The growth of POI data and its usage creates opportunities for revisiting and generating new research questions. POI research questions should address geospatial and social representations, functions, and implications of individual POI instances, types (i.e., POI categories such as “clothing store” or “football club”), aggregates of the same types, and agglomerations of different types. These questions connect POI relations to people, places, and human settlements. This section sets out scientific and societally relevant questions that can be addressed using digital POIs.

### Questions related to places

Place is a core concept that has long been widely discussed in human geography, urban planning, digital humanities, cartography, and GIScience (Agnew, 2005; Goodchild, 2011; Janowicz, 2009; MacEachren, 2017; Purves et al., 2019; Tuan, 1977). Place is not only a geometric point, but also reflects interactions among locations (*locality*), the linkages to daily social activities (*locale*), and individual/community sense of place (Agnew, 2005). How does a POI location become a place and for whom? Is it determined by the activities the POI affords or by the time and frequency of people’s visits and interactions with the POI? How does a POI’s surrounding context influence its functions, clientele, and activities? And what about the longevity of a place (i.e., how old or new it is and how much it has changed)? How could the POI longevity aspect contribute to an understanding of what neighborhoods are changing to?

### Questions related to people

POIs serve people and can reflect the characteristics of the communities they serve. With the increasing coverage of POI data (i.e., more POI records in more places) publicly available for use and wider dimensionality of accompanying attributes (covering spatial, temporal, and social aspects of POIs), new avenues open up to the study of interactions between people, places, and people and places (Janowicz et al., 2019; Psyllidis et al., 2018; Silva et al., 2013). Such questions include:

*Who uses what POIs? Are certain groups at an advantage and others at a disadvantage in the landscape of POIs?* Demographic (i.e., census) data can answer who is proximal to a POI, and we can further examine GPS trace data to see who is actually visiting the POI. While POI owners (e.g., a concert venue) tend to have information about who visits their venue (e.g., via credit card or survey information), the public may not be able to access that data. Findings from scientific literature or common knowledge about POI usage can be used to ascertain who a POI may be serving: e.g., temples, mosques, or senior centers may have clientele that can be (sometimes) inferred. Another method for determining who uses POIs is to use geo-tagged/geocodable posts from social media to gather information on who is sharing their experience through photos or text reviews. In some cases, when we detect a preponderance of POIs that are accessible, affordable, and draw/serve certain groups (such as tourists or teens), we can suggest that this group is being well-served by the affordances of the built environment.

### Questions related to human behavior (activities)

How and why do people use POIs? How do POIs co-evolve with technologies and cultural changes (e.g., a shift from hotels to AirBNBs) that drive demand? How do the activities that occur at POIs contribute to outcomes such as innovation, disease transmission, economic activity, and crime? Is insufficient access to amenities associated with worse outcomes overall or higher levels of urban inequality?

*How does the density, diversity, and configuration of POIs affect activities of urban life or urban inequalities?* How are POI visit patterns different between local residents and visitors? POIs may allow people to mix who may not be mixing through serendipitous interaction that prevents segregated neighborhoods or income-segregated workplaces. subsequent encounters with other population groups (e.g., of different ages, ethnicities)?

### Questions related to effects of POIs

What are the benefits and drawbacks of living near different POIs? There may be burdens associated with living near (or being exposed to) POIs. These could include measurable features such as noise, environmental degradation (pollution, smoke, unclean drinking water), and quasi-measurable features such as associated crimes (Groff & McCord, 2012). The placement and presence of some POIs are politicized and spur not in my back yard (NIMBY)-related effects, as neighbors oppose plans to place POIs near their homes. These can include nightclubs, gas stations or liquor stores, or social services such as Planned Parenthood or methadone clinics, despite evidence that their placement does not lead to increased local crime (Gupta et al., 2012). There can also be fringe benefits of being near a POI, such as proximal jobs, a jogging track at a high school, or free public lectures near a university.

*Who is served by a POI and who benefits?* Is it loud, is it accessible for people with disabilities or mobility impairments, is it appropriate for children, etc.? Is the setting intimate (small crowds) or is it a large venue?

*What are the “spillover” effects of a POI?* POIs have inherent agglomeration effects, meaning that a set of many POIs may be clustered. Sometimes these locations are planned (e.g., a shopping center), while other times, a POI will choose a location based on the existing landscape of POIs (e.g., a new restaurant opens near a metro station or a bar opens outside of a theater), resulting in the emergence of new POIs.

## POI data sources, attributes and dimensions

### Data sources

POI data can be obtained from two general types of sources: big-tech companies and open-source platforms. Big-tech companies, such as Yelp, Foursquare, Google Places, and Facebook, generate rich amounts of POI data, and similar prominent companies include Baidu and Gaode Maps (in China). OpenStreetMap (OSM) has provided POI information that is free and available worldwide, though with varying levels of completeness (Barrington-Leigh & Millard-Ball, 2017) and with many corporate contributions (Anderson et al., 2019). Additional sources include Niche.com, Wikimapia, TomTom, and HERE map, OneMap, or POIs that are provided by mobility data companies such as SafeGraph and Cuebiq. Many datasets are accessible through Application Programming Interfaces (APIs).

Big-tech companies create and maintain POI data in order to provide location-based services to users (e.g., recommending nearby restaurants based on the current location of the user). While most companies keep their POI data internally, companies may openly share part or all of their POI data for non-commercial use (e.g., https://www.yelp.com/dataset). POI data sources on open-source platforms such as OSM and Wikimapia are maintained by users, who contribute and volunteer time to creating new features in these platforms, and editing current information, including “hobbyists”, “mappers”, and “fixers” with systematic or casual editing frequencies (Quinn, 2017). Anderson et al. (2019) find that over half of all edits to OSM are from corporate entities, blurring the lines between traditional user-generated content and corporate interests. Despite the many eyes that govern these datasets, POI data on these open-source platforms may still have coverage and data quality bias issues (e.g., certain geographic areas are better represented than other areas). However, a major advantage of these POI data is that they are open and can often be used for both non-commercial and commercial purposes when credits are properly attributed.

### Attributes

POI datasets include, foremostly, the POI name, its type, geospatial coordinates (typically a lon/lat point, and less often a building footprint), business hours, contact information (e.g., website), and potentially — depending on the source — number of user reviews, average review rating (e.g., 5 out of 5 stars), information about prices, photos, among others. The latter tend to be user-generated content. POIs are categorized within a typology. POI typologies are data structures that are typically pre-labeled and organized into high-level categories (e.g., health, services, food/dining, recreation) followed by lower-level categories (e.g., Chinese food) and further sub-categories (e.g., Hot Pot). Because these structures often accommodate bottom-up POI types (that is, a user provides a new POI and enters in a new type, or a business is registered with a new type and becomes part of a dataset), we can find new venues such as “Bubble Soccer”, “Ax Throwing”, and even “Cuddle services” on platforms such as Yelp or Foursquare.

#### Name and type

POI types play a prominent role in applications such as online mapping, social media, and tourism. Since a POI represents a location labeled by individuals, it follows that the POI name is a unique value or an affordance based on an activity, resource, etc. In short, many places, at least in urban environments, exist because some activity is conducted at that location (e.g., eating at a restaurant, walking in a park). In all cases, these activities include additional dimensions which are inherited by the POI and typically represented through a communally agreed upon place type or activity label (e.g., clothing store, police station).

#### Geometry and positioning

The canonical set of vector data, including points, lines, and polygons still presents a set of options for POI representation, despite the nomenclature of ‘point’ in POI. That said, POIs are most often represented as *point* data (de Graaff et al., 2013). While points are a useful — but often inaccurate — representation of places, they have become the de facto reference format for navigation, representation, and analysis POIs.

**Fig. 1:**
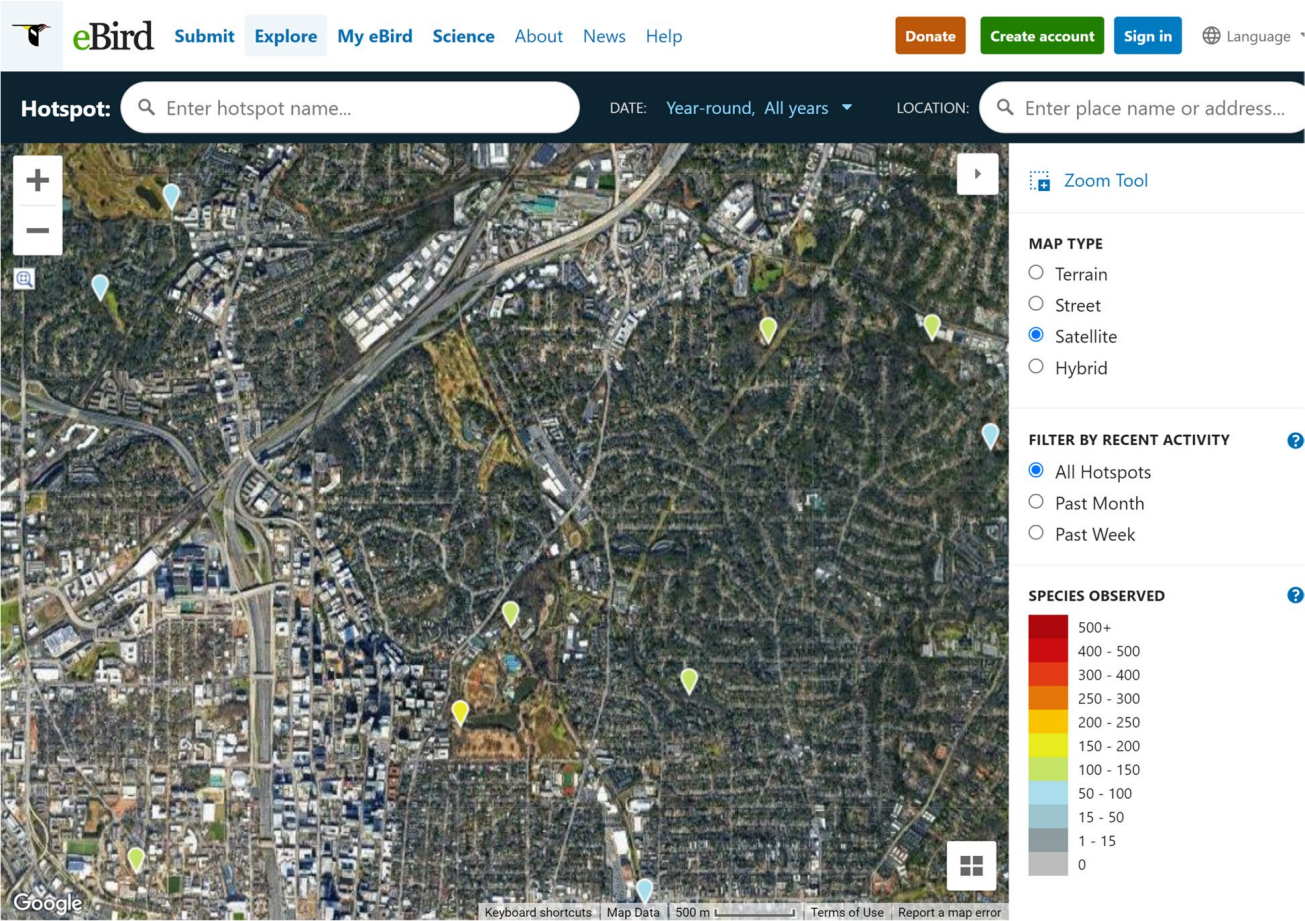
A screenshot from ebird.org in Piedmont Park, Atlanta, Georgia, U.S.A., Although birders detect birds throughout the park, there are only two options to geolocate their findings (one at the park information desk, and one at the botanical gardens information desk). This could lead users to believe that birds are concentrated in those two areas

Certain POIs work well as points, such as a street kiosk (news shop), which has a small footprint and has only one access point (via the sidewalk). Other POIs should be represented as *polygons* that can express the size and shape of the feature and can communicate that the POI is spread across an area. Without the polygon representation, fidelity can be lost. For example, at ebird.com, users create digital information of bird sightings. Users must choose a POI point to identify the location of their sightings. However, the birds are actually discovered over a wide swath of area (Fig. 1); perhaps none exist at that exact point they are assigned to.

**Fig. 2:**
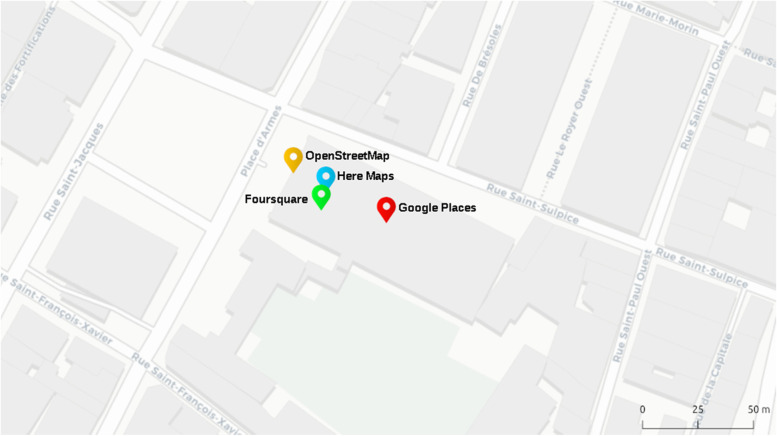
Four POI-based digital representations for the same real-world location (Notre-Dame Basilica in Montreal). Each point is labeled by the POI provider. Base map by CARTO/OpenStreetMap Contributors

Neglecting to express POIs as polygons can also affect populations who rely on information on accessibility. When a POI such as a park or a department store is represented as a point, the access points into the POI are usually unknown. Users who may rely on accessible entrances or require an accessible curbside drop-off, may not be able to gauge the difficulty of entering a building. This can be further reinforced by distance differences in a POI’s location between different sources (McKenzie et al., 2014) (Fig. 2). Knowing more about whether a POI is offset off a road, and where the entrances are can help visitors plan their trip or choose a destination from a set of potential POIs.

The geometric representation of POIs may further influence the way we assess how accessible given facilities and other amenities are, which can subsequently impact planning and public health policies and interventions. A prominent example concerns the way access to green (and other outdoor public) spaces, such as parks, is measured. The entrances connecting parks to the road network are often overlooked, and access is almost exclusively measured from either the park centroids (i.e., the geometrical centers) or — in the case of polygons — from points along the park edges (Halden et al., 2000; Wang et al., 2021). However, polygon centroids are abstract simplifications that barely correspond to how locations are actually accessed and, further, not every point along a park edge is accessible from the road network. These could introduce substantial bias to the measurement of accessibility, especially in the case of large parks and other outdoor public spaces (Talen, 1997; Weiss et al., 2011). In closing, we emphasize the importance of accounting for the context of use of POIs in considering both forms of representation (e.g., as points or polygons) and the precision required of such representations.

#### Temporality

One of the most informative POI dimensions is time, or rather the temporal patterns of activities that occur at the POI. For POI types such as businesses, these temporal patterns may take the form of “hours of operation”, as socio-institutional constraints limit the ability for a visitor to attend a POI outside of operating hours (Raubal et al., 2004). In other cases, such as public squares, such an institutional construct does not exist, meaning that temporal patterns are only limited by the accessibility and interest of the visitors.

As is the nature of any representation in GIScience, POIs are an abstraction of a real-world place. The times of day, days of the week, months of the year that activities occur at a place have their real-world patterns, and then there are the representations of those patterns available to researchers and individuals via data collection. Today, data collection for activity patterns at POIs takes on two forms: (a) *active* data collection and (b) *passive* data collection. Surveys of individuals at places or geosocial media check-ins are examples of active data collection, where an individual is aware of their data being collected or contributed. On the other hand, visitation patterns as determined through context-aware mobile device sensors, mobility tracking, and place inference are examples of passive data collection. Both of these options present perspectives on the real activities being conducted at POIs. Often, these two methods for representing real-world temporal patterns of POIs present very different perspectives. It could be argued that passive data collection best represents actual visitation patterns as users are not selecting which place types to share data and which not to share data. By comparison, active data collection (e.g., check-ins) reflects socially salient behavior. One is more likely to share their visitation to a trendy night club than the gastroenterologist.

Temporal patterns vary in scale as well. While the hourly/daily variability of a popular dance club may significantly contribute to its place type label (i.e., dance club), a war memorial’s temporal visitation pattern may be more descriptive at an annual scale. There are seasonal effects to temporal patterns that vary by geographic region (McKenzie et al., 2015). The importance of scale is also apparent in which temporal patterns are reported. If temporal behavior is reported, it is often reported hourly or daily. Depending on the use-case for the data, this may or may not be sufficient.

Moreover, POI locations can change over time: a business can move to a new location, a POI can close or a new POI can open. On the user end, platforms like Google Maps and Yelp list information that a POI is permanently closed, but not that a POI is new, thereby providing a one-sided view of temporality. Because turnover can be prevalent, POI datasets should be updated frequently, and POI providers should be able to refresh data accordingly. The changing landscape of POIs can provide an opportunity for new research questions about (a) which POI types tend to have longevity or are less resilient (to changes in economic or political circumstances, or natural disasters), (b) which places and environments are experiencing POI-related growth, decline or turnover, (c) what circumstances lead to these outcomes. Updated POI datasets are important also because they can be used to signal positive or negative changes in what is accessible to residents. For instance, food deserts (i.e., neighborhoods where it is difficult to buy affordable or good-quality fresh food) and food swamps (i.e., neighborhoods with an abundance of non-nutritious food)  are often prominent in disinvested neighborhoods (Cooksey Stowers et al., 2020), which are often home to disadvantaged groups. POI datasets can track the availability of healthy, proximal food options over time, meaning that researchers can see whether neighborhoods used to have healthy options, but no longer, whether healthy options are becoming more abundant, or whether there has been little change. The trajectory can help us identify neighborhoods that are in need of intervention. 

Lastly, some POIs do not have typical temporal patterns as visitation by people does little to contribute to their definition as a place type. For instance, a mountain top exists as a POI due to its physiography rather than the way in which people interact with it. Compare that to a nightclub, whose identity as a nightclub is highly dependent on the temporal visitation pattern. It is important to realize that the significance of the temporal dimension in contributing to the value of POI varies based on geography as well as type.

## Applications: past, current, and future

In this section we outline how POIs are used in research and practice.

### Health (physical and mental)

A growing body of evidence has highlighted the effects of particular built-environment features, such as street connectivity, distribution, density and mixture of land uses, on promoting (or obstructing) healthier lifestyles and improving (or degrading) the well-being and quality of life of inhabitants (Cerin et al., 2017; Giles-Corti et al., 2014; Hooper et al., 2015; van Holle et al., 2012). Availability, proximity and accessibility to specific POIs and, most importantly, the ways that people interact with their surrounding POIs can lead to different health outcomes. For instance, exposure to outdoor greenspaces, such as parks and regular visits to healthy food stores have been associated with increased physical activity, lower levels of obesity and improved levels of cognitive development and mental health (Coombes et al., 2010; Garcia et al., 2020; Labib et al., 2020; Larson et al., 2009; Sugiyama et al., 2010; Zhu & Fan, 2018). Contrariwise, interactions with some other POI types, such as liquor stores and tobacco shops, may lead to negative health outcomes (e.g., excessive drinking and intoxication, high blood pressure) and social issues (e.g., violent behavior) (Chang et al., 2022; Phan et al., 2020). Information about the types of POIs and the frequencies and time durations that people interact with POIs can help us further understand the potential links between the use of (and exposure to) POIs and related health outcomes. Besides direct interactions, exposure to proximal POIs may also have health implications. Health can be affected by exposure to particular attributes of POIs that have restorative (e.g., lower anxiety) or harmful (e.g., air or noise pollution) effects, without necessarily visiting these places. For instance, visible blue spaces (e.g., canals or lakes) from one’s residential space were associated with lower psychological distress (Nutsford et al., 2016). Contrarily, outdoor advertising of food and beverages in residential areas has been associated with increased obesity levels among the residents (Lesser et al., 2013; Wray et al., 2021).

### Mobility

POIs have been an emerging data source for mobility studies at both population and individual levels. Attributes pertaining to popularity or visiting patterns indicate floating population size visiting a POI within a given time period. For instance, Google Maps represents POI popular times through statistically estimated and real-time visiting population sizes (e.g., via average or real-time mobile phone signals, respectively) on each hour and day. Such ambient population information on a fine spatiotemporal scale can benefit research on understanding city-level dynamics of population and mobility. POI-based floating population data are semantically richer than cellphone based data provided and monetized by telecommunication companies. Even though the accuracy of a mobile phone user’s location tracking has been improved through GPS and WiFi positioning on top of cell tower triangulation, uncertainty exists in inferring which place is actually visited by the user, especially in city centers and buildings with a multitude of shops and services. In contrast, POI-based data such as popular times on Google Maps utilize both location tracking and search history for places obtained from millions of users, which may enable more accurate inference of a user’s POI visits (D’Zmura, 2020). Such POI-based mobility data have become useful to understand dynamic urban phenomena and apply practices in public/private sectors regarding daily mobility behaviors, transport engineering (e.g., taxi demand prediction; Askari et al., 2020), infectious disease dynamics (e.g., COVID-19 transmission; Psyllidis et al., 2021; Zhang et al., 2022), tourism planning (e.g., Shi et al., 2020), disaster management (e.g., Podesta et al., 2021), and public service optimization (e.g., predictive policing; do Rêgo et al., 2020).

### Development of human settlements

Recently, the United Nations (UN) published the 2030 Agenda for Sustainable Development, listing the pathways towards healthier, safer, and more inclusive human environments (United Nations, 2016). Specifically, regarding the resilient and sustainable development of human settlements (i.e., Sustainable Development Goal, SDG11), a number of targets and corresponding indicators to evaluate their achievement have been developed. Indicative examples include safe and inclusive access to amenities, such as public transport systems, green and public spaces (targets 11.2 and 11.7, respectively), and the fostering of participatory and integrated human settlement planning (target 11.3). Relevant indicators revolve around metrics, such as the proportion of population having convenient access to public transport and greenspaces and average shares of built-up areas. From a POI perspective, these SDGs call on an inquiry into the potential role of POI data in building customizable and context-specific indicators that account for different age and societal groups, people’s needs and preferences. Moreover, it remains to be explored whether the availability of reliable POI data can foster the capacity for participatory, integrated, and sustainable human settlement planning.

### Urban and regional planning

In urban and regional planning, the POI amenities in shopping centers, downtown areas, and rural areas should suit the needs of the local population. There is no one-size-fits all for the perfect POI mixture, and the decisions on what to put where is often proprietary information codified by land developers who may or may not prioritize the best interests of the people over profits and markets. But can we measure how well a set of POIs is helping the people around them? We often measure distribution, density, and mixture of amenities at scale (Hidalgo et al., 2020; Song et al., 2013), which can detect evidence of classical location theories of business agglomeration (e.g., Sevtsuk, 2014), and can help evaluate equitable distribution of resources (Logan et al., 2021; Sheriff & Maguire, 2020). Newer methods use semantics to detect variation and sets of POIs that are commonly found together, e.g., a bookstore and a coffee shop (Liu et al., 2019, 2020, but examples abound). Sometimes a homogenous set of POIs cluster, while other times, heterogeneous sets of POIs cluster. Such papers are more focused on pattern recognition (and arguably its roots with older economic geography theories, as mentioned above), than how the places serve the people, and who benefits, and what should be there instead. Finally, in terms of economic development and generating wealth for communities, POIs can help cities understand how consumers are spending on tourism, retail and food, and what districts have more or fewer opportunities for these expenditures.

### Urban analytics

While individual POIs provide specified services, POI collectives reflect the nature of a community, residents, and prospects. Studies used POI types and frequencies to measure urban vibrancy (Yue et al., 2017), model the distributions of urban functions (Huang et al., 2022), delineate urban functional zones (Gao et al., 2017; Liu et al., 2020; Psyllidis et al., 2018), or identify urban lifestyles (Li et al., 2020). Urban functions can be extracted by mining co-occurrence of POI types and by integrating human activities on location-based social networks (Gao et al., 2017).

### Community studies

While less studied, POIs correspond well to the demographic and socio-economic characteristics of the residents and neighborhoods whom the POIs serve. Studies suggest connections between POIs and people. Dollar stores and fast-food POIs populate around lower-income neighborhoods (Dhakal & Khadka, 2021). Culturally unique and authentic restaurants are associated with neighborhood wealth (Yu and Margolin, 2021). A healthy community has easy access to trails, parks, and other green or blue infrastructure. The presence of Asian restaurants or stores suggest clusters of Asian populations. In contrast, if Asian, Mexican, Indian, and Muslim grocery stores are distributed in a mix across a city, we may expect more racially diverse neighborhoods. Districts with more senior/elderly residents are likely to have more stores and services tailored to senior customers. Neighborhoods around primary schools are likely to have access to stores and facilities for kids or families, much different from those around colleges. Communities should encourage Social Infrastructure Mapping activities, wherein a community takes inventory of how their POIs serve as public, private, or hybrid meeting spots, whether they have enough POIs and whether these are accessible. This is another way that POIs can support a healthy community, since relationships and strong social life is a key factor in mental and physical health (Cacioppo & Patrick, 2008). Through events and serendipitous interactions (e.g., running into a friend at a farmer’s market), communities should take stock of how many instances are available for people to interact (Mehta, 2014; Mehta & Bosson, 2010; Rosenbaum, 2006). Communities that are missing these places should prioritize programming infrastructure (streets, buildings) to support social life, and consider who in the community will benefit (teens, elderly, families, romantic couples, etc.).

### Sociology

In social capital literature, POIs are often referred to as ‘institutions’, and are used as proxies for social capital in the built environment. One Social Capital Index at the U.S. county level (Rupasingha et al., 2006) equates the presence of *gyms, bowling alleys, golf courses, businesses, and religious institutions* with high levels of social capital. This means that areas with more of these specific POIs are deemed to be better for creating relationships that provide resources. Sociologists also use the concept of ‘memberships’ to measure social capital and civic engagement, such as membership to *veteran’s groups, religious groups, women’s groups, political groups, school groups, hobby groups, neighborhood associations,* etc. (Lee & Kim, 2013) and it is unclear whether these are brick-and-mortar institutions and or virtual or conceptual memberships.

That said, equating social livelihoods with institutional membership is both good and bad for the study of POIs. First, it is good because showing that POIs help social interaction provides new evidence that the built environment affects quality of (social) life. However, it presents a problem because it is unknown whether individuals need to be near POIs (i.e., if POIs need to be placed in areas that are accessible to households) for individuals to benefit from them. Another problem with linking the presence of POIs to social livelihoods is that we do not know whether people actually interact with or are members of the POIs that are near them. Therefore, GIS analyses of accessibility to POIs are incomplete because having a POI (like an expensive country club) near a neighborhood does not mean it is useful to that neighborhood.

## Challenges

### Engineering and analytical challenges

#### POI matching and updates

POI data collection, update, matching, conflation, database management and analytics are important building blocks in the POI data pipeline. Inconsistencies in the geo-location and classification of POI types can substantially affect measures of accessibility, land-use mixture, and segregation, with considerable effects on the resulting policies and interventions. The real-world place entities are dynamically changing over time (e.g., restaurant closure or re-opening). How to maintain an accurate and most updated POI database is a grand challenge. Great efforts have been made in POI and gazetteer matching and conflation of existing POI databases using various methods such as multi-attribute weighting (McKenzie et al., 2014), graph-based matching (Novack et al., 2018), and deep neural networks (Santos et al., 2018). Given the availability of street-view imagery, web documents, and deep learning advancements in computer vision and natural language processing, future studies may consider integrating multi-source and multi-modal data (including spatial, temporal, visual, textual information) for POI matching and update. Hierarchical relationships of POIs can be automatically constructed using spatial topology, such as defining a parent–child relationship between places if a POI is contained by an overlapping polygon. This is particularly important for capturing POI types with closely related characteristics (e.g., beauty salons and hair salons). The prevalent top-down POI type hierarchies often inhibit the identification of such similarities, and recent research approaches advocate the use of place type embeddings, which are analogous to the word embeddings used in linguistics (Jin et al., 2019; Yan et al., 2017; Zhai et al., 2019).

We can address these issues by providing a small buffer zone around a building footprint and aggregating points that fall within the footprint or the (admittedly subjective) buffer zone around the footprint. Footprint data, however, may not be appropriate for certain elements (like signs), or may be difficult to acquire. In these cases, the geographic mean or median of the location coordinates can be used as a consensus point, or a bounding envelope of the points (if there are three or more) can be used to provide an area of confidence for the POI location. To assess error across POI datasets comprehensively, traditional spatial statistics like Ripley’s *K*-function can show variation in positional accuracy (Yeow et al., 2021). An alternative method is to create composite keys (multi-attribute matching) from sets of attributes in datasets (see Deng et al., 2019).

#### POI labeling and categorization

Oftentimes, several POIs either lack labels about their category or are erroneously categorized (e.g., a nightclub classified as a cultural space). Commonly, machine learning (ML) classifiers are employed to label POIs with a place category (Choi et al., 2020; Giannopoulos et al., 2019). Pre-labeled POI data from a single or multiple data sources serve as the baseline for the categorization of unlabeled or mislabeled POIs. The process involves extraction of features from annotated POI data and their representation as feature vectors, which are used to train the labeling classifier. Common approaches to POI classification use either several or limited features (Lu et al., 2020; Choi et al., 2020). Recent evidence suggests that different features have a varying influence on the classification results (Milias & Psyllidis, 2021). In particular, features pertaining to the operating hours of a POI appear to be more indicative of its category relative to features pertaining to visitation patterns or surrounding context. Nevertheless, there are fluctuations depending on the POI category. For instance, spatial configuration plays an important role in categorizing POIs that tend to cluster, such as retail stores or cafés (Janowicz et al., 2019; Milias & Psyllidis, 2021).

#### Spatial analysis and scale

Additionally, without comprehensive POI data, the results of operations such as point pattern analysis can change. If an analyst is trying to find whether points cluster, for example, a dataset that has too few records can yield statistically insignificant results, when in reality there may be a significant cluster of points at a location. If these POIs are not present in the data, the conclusions may not reflect ‘reality’.

One major research challenge with POI analysis is where to draw lines of a study area that includes POIs, and how to deal with edge effects. This is a common issue with aggregation of points in general, as, first, changing the geographic scope of analysis will change spatial analysis statistics (including first and second order point pattern analysis and variety statistics). This also happens when the geographic scale of analysis changes, for instance, from county scale to state scale. Second, excluding edge cases, i.e., POIs that fall outside the study area but still serve those inside the study area, can lead to under or over-representing individuals’ access to amenities. The optimal scale can be perhaps calibrated by buffering a study area with commonsense travel distance, or through sensitivity tests. In addition, when using POI features (e.g., counts, categories, prices, reviews) combined with machine-learning models for urban neighborhood characterization, the model performance may vary across different geographical scales (spatial resolutions) and across cities with varying population sizes (Dong et al., 2019).

#### Analyzing accessibility

Various measures of walkability and accessibility have been developed over the years (Clifton et al., 2008). Conventionally, the accessibility of different POIs and the degree of walkability of neighborhoods containing a number of POI collectives are evaluated either according to the distribution of POIs within a given area (e.g., administrative boundaries of a neighborhood or a city) or by means of circular buffer zones, usually around the residential space (i.e., individual households or the centroid of residential areas) or neighborhood center (Oliver et al., 2007). Such approaches often disregard the dependency of human access on the road network and the chosen transport modality. Moreover, activity behaviors and social interactions of non-working populations (i.e., children and the elderly) at POIs are barely considered in these measures, usually because of scarcely available data on the activity patterns of different age groups (Xu, 2019). A more recent — and, thereby, relatively disregarded — aspect of accessibility pertains to ‘virtual’ or non-brick-and-mortar POIs. Digital places like RideShare, Grocery Delivery, and Online Retail means that many services are available to people, but will not be found on the map. Therefore, not living near public transportation or a grocery store does not necessarily mean that households cannot access transportation services or food. In some cases, POI type prevalence correlates with demographic information (Dong et al., 2019), and can be used as a heuristic for measuring who might have access to POIs (assuming that proximity indicates access).

### Information and communication challenges

#### User-generated contribution

POI data construction and maintenance on both open-source and commercial platforms heavily rely on people’s direct (e.g., OpenStreetMap) or indirect (e.g., GoogleMaps) contributions of spatial/platial information. Critical to such crowdsourcing initiatives is the design of effective incentive mechanisms to motivate people’s participation (Morschheuser et al., 2019). Incentives may either be extrinsic, such as monetary and other utilitarian benefits, or intrinsic such as altruism, the sense of accomplishment, learning, and relatedness with peers have often been adopted (Budhathoki & Haythornthwaite, 2013; Morschheuser et al., 2019). Building contributor communities is one of the approaches that can stimulate volunteerism in a local community (e.g., mapathons for humanitarian mapping after global natural hazards; Dittus et al., 2016). Gamification has also been employed to invoke competition or cooperation among contributors (Morschheuser et al., 2019). An indicative example is Google Local Guides, which was recently launched by Google Maps and leverages an online contributor community, gamification features including a point system, levels, badging, and user awards to encourage users to contribute more content to Google Maps (Local Guides Help, 2022). To ensure data quality, it would be crucial to draw data donations from major map content creators such as local governments and professional mapping agencies. Yet, the fragmented nature of the population groups contributing information to social media (i.e., predominantly young, affluent, urban populations) relative to the wider society, introduces considerable bias to the nature of the extracted interactions. Even though, in reality, poor and vulnerable social groups appear to use public spaces more than high-income residents, little is known whether this is also reflected in online interactions at given POIs.

#### Gender classifications

Deeming POIs as masculinized or feminized in practice without gray area between the spectrum can affect research findings about knowledge production. Das et al. (2019) find that women OSM editors tend to frequently edit masculinized spaces and vice versa: *“85.90% of male contributions involved feminized spaces, while only 68.18% of female contributions involved those same *types* of spaces. Alternatively, masculinized spaces received 31.82% of female edits, but only 14.10% of male edits.”* (Das et al., 2019, p. 8). In this analysis, the authors use a classification derived from prior work that classifies POIs such as ‘nightclub’ as *masculinized* spaces (Scanlon, 2007), which in practice, may be a fun entertaining space for both genders. The top ‘gendered’ spaces mapped by female editors was the nightclub (18) — more so than childcare, kindergarten, nursing home, etc. Conversely, males mapped kindergarten (2,388 times), and nightclubs (634 times). We note that these edits are on very different orders of magnitude. Still, these results may lead us to question the benefits and perspectives involved in associating a POI with one gender as a theoretical principle (caretaking vs. sexual pursuits). It may be helpful to also capture gender proclivities by gathering anonymized information on who visits and works at these venues and who considers these to be important.

#### POI visualization and interfacing with the people

Our ‘reference’ maps now have retail and for-profit icons, which are different than before. People are used to having base maps online and as they explore and search for things in the built environment, we should be aware of things like advertising and decisions for prominence (i.e., what do we see first?).

## Prospects for the future and conclusions

The major priorities for POI research and use in the future are ensuring data completeness and encouraging reflection on how POIs address human needs.

Regarding data completeness, we recommend a panel (i.e., longitudinal) dataset on POIs, and that researchers consider what an *ideal* dataset would resemble, and how to foster a true OpenPOI dataset where anyone with a mobile phone can make additions or changes easily. This would allow for more democratization in the POI process and allow for wider spatial coverage. POI datasets should be available to the public for free and updated frequently. We also can benefit from ontological design patterns for determining what should be considered a POI and what should be considered just spatial data (e.g., sidewalk, or home). Currently, OpenStreetMap, the gold standard for open spatial data, only allows for point-based spatial data with regards to POIs. Entries are relatively difficult to update, and have trouble accommodating name changes, type changes, positioning calibration (e.g., how offset off a road a POI is, or changes in the location of entrances), or monthly changes such as a new wifi password at a coffee shop (McKenzie & Janowicz, 2018).

In the future, we should model how the POIs help people and what their utility is. POI utility depends partially on the events that occur there, colloquially known as ‘programming’. For example, a library may offer yoga for the elderly, or a church may have Alcoholic Anonymous meetings — both are good for the wider community and add to the value of the POI past what we would normally think their ‘target demographic’ may be. We lack information about the activities that occur at POIs. When a POI hosts events and engages social connections, the POI becomes a place that embeds social and cultural contexts and infrastructure functions to support human activities and shape social relationships. For example, communities with parks facilitate Easter egg hunts or block parties which strengthen familial and neighborhood relationships. The interdependency between place and social relationship can lead to geographic inequity of both physical and mental health outcomes (Kane & Margerison-Zilko, 2017). A place or facility to host festivities can bring good will to a community and offer opportunities to build new networks or revitalize existing ones via social capital (Mair & Duffy, 2020).

In conclusion, we believe that universal access to high-quality and reliable POI data is key and that public and private players in the POI data ecosystem should work toward these goals. Rich and publicly available POI data would contribute to a better understanding of the complex interlinkages between people and places, as described in the scientific questions outlined above. Finally, a more democratized generation and use of POI data could contribute to planning and designing context-sensitive policies and interventions towards liveable, resilient, and healthy communities.

## Data Availability

Not applicable.

## Footnotes

1 This event was free and open to the public. However, participation was limited to those who saw the notice of the event through mailing lists or public social media posts. It was held during Eastern Time business hours, which was not accommodating to a global audience
